# Nanoparticle-enhanced cytology: a 2025 breakthrough for rapid diagnosis of Erdheim–Chester disease

**DOI:** 10.1097/MS9.0000000000004324

**Published:** 2025-11-14

**Authors:** Awais Butt, Araj Naveed Siddiqui, Maliha Khalid, Muhammad Talha, Aminath Waafira

**Affiliations:** a Department of Medicine, Aziz Fatima Medical and Dental College, Pakistan; b Department of Medicine, Jinnah Sindh Medical University, Karachi, Pakistan; c Department of Medicine, King Edward Medical University, Lahore, Pakistan,; d Department of Medicine, The Maldives National University, Malé, Maldives

**Keywords:** Erdheim–Chester disease, nanoparticle cytology, rare disease diagnosis, surface-enhanced Raman spectroscopy

## Abstract

Erdheim–Chester disease (ECD) is an ultra-rare non-Langerhans cell histiocytosis characterized by CD68-positive, CD1a/S100-negative histiocytes, frequently associated with BRAF V600E and MAPK pathway mutations. Diagnosis remains challenging due to its nonspecific presentation, reliance on invasive biopsies, and frequent diagnostic delays of several years. Nanoparticle (NP)-enhanced cytology, including surface-enhanced Raman spectroscopy, represents a promising breakthrough in 2025, enabling single-molecule sensitivity and multiplexed biomarker detection on cytology slides. NP-based imaging agents, such as Cu-Macrin for positron emission tomography, demonstrate high specificity for macrophage-rich lesions, while NP assays improve capture of circulating tumor DNA, including BRAF V600E. When integrated with AI-driven image analysis, NP-based approaches may overcome current diagnostic barriers, enabling earlier, less invasive, and more precise identification of ECD. Standardization of NP-based diagnostic protocols and continued research are essential to validate these emerging technologies and translate them into clinical practice.

*To the Editor,*

Erdheim–Chester disease (ECD) is a rare non-Langerhans cell histiocytosis classified as a neoplastic hematopoietic disorder, characterized by CD68-positive, CD1a/S100-negative and foamy histiocyte infiltrates in multiple organs, such as the retroperitoneum, cardiovascular system, central nervous system (CNS), and long bones. Involvement of these critical structures, such as the periaortic region, right atrium, and CNS, often prompts investigation^[1,2]^. However, the disease shows no early symptoms, which results in delayed diagnosis. ECD is associated with the genetic mutations in BRAF V600E and MAPK, which are ultimately related to several types of cancers (pulmonary adenocarcinoma, melanoma, etc.), making ECD a malignant disorder. The disease shows both inflammatory and neoplastic symptoms, making its pathophysiology difficult to understand. ECD is extremely rare. It tends to occur in one in a million individuals, providing insufficient data on its exact prevalence. The rarity of the disease produces significant diagnostic challenges and offers a limited understanding of its nature^[1]^.

Current diagnoses depend on tissue biopsy, which may be taken from bone or organs. It requires histology and immunohistochemistry analysis for interpretation of the results, making it invasive and inconclusive. This often delays treatment by 2–5 years due to subtle early signs, particularly in neurological and meningeal presentations. The diagnosis of ECD is often postponed due to the presence of non-specific histiocytic infiltrates in cytology samples. Current cytomorphologic methods, although valuable, tend to produce samples with non-specific histiocytic infiltrates, complicating differentiation from other histiocytic disorders^[3]^. A more recent technology, NP-enhanced cytology, such as surface-enhanced Raman spectroscopy (SERS), may offer a transformative approach to the accurate diagnostic sensitivity to a single molecule level and reduce procedural invasiveness in ECD. While not specific to ECD yet, this emerging method may enhance diagnostic precision and may improve the approach in future applications^[4]^.

Recent studies in NP-based imaging, such as Cu-Macrin (a 20-nm poly-glucose NP), have been developed for quantitative positron emission tomography (PET). It is estimated that it can achieve over 90% specificity for macrophage-rich tissues in preclinical animal models^[4]^. These NPs, due to enhanced permeability and retention effect, tend to settle in histiocytic lesions. Recent radiologic reviews of ECD suggest 18F-FDG-PET for detecting glucose metabolism regulated by BRAF-driven Glut-1 expression in ECD histiocytes, distinguishing it from other types of histiocytosis^[2]^.

Recent studies have shown that NP-based assays can significantly improve the capture and detection of circulating tumor DNA, including BRAF V600E mutations. This advancement improves non-invasive diagnosis that could enhance the robustness of future ECD findings^[3]^.

Additionally, as a newly developing technology, SERS can be multiplexed to detect multiple ECD biomarkers, such as CD68, BRAF V600E, and Glut-1, simultaneously on a cytology slide. This approach could make the diagnosis of ECD relatively easier^[3]^.

To conclude, NP-based cell analysis marked a significant breakthrough for ECD diagnosis in 2025. Notably, AI-based image analysis with NP-based assays and liquid biopsies has shown potential to optimize the diagnostic pathways. These technologies could facilitate early ECD diagnosis. We therefore recommend that the pathology societies establish standardized protocols for NP-based techniques and conduct more research in emerging yet highly valuable technologies that can enhance the findings in ECD. This letter to the editor is in compliance with the TITAN guideline^[5]^.

## Data Availability

No data were generated for this manuscript.

## Research registration unique identifying number (UIN)

1. Name of the registry: N/A. 2. Unique identifying number or registration ID: N/A. 3. Hyperlink to your specific registration (must be publicly accessible and will be checked): N/A.
